# Assessment of current use pesticides in flowers, pollen provision, and wild bees: HPLC-ESI-MS/MS method development and field implementation

**DOI:** 10.1007/s00216-025-05935-8

**Published:** 2025-06-11

**Authors:** Carolina Honert, Katharina Wifling, María José Lazo Hernández, Carsten A. Brühl

**Affiliations:** iES Landau, Institute for Environmental Sciences, University of Kaiserslautern-Landau, Landau, Germany

**Keywords:** Pesticides, Agriculture, Insects, Contamination monitoring, HPLC-ESI-MS/MS

## Abstract

Synthetic pesticide use is a major driver of pollinator declines in agricultural landscapes. To understand the impact of pesticides, it is essential to quantify residues in food resources and in insects themselves. We developed simple, fast, and cost-effective multiresidue methods for the simultaneous quantification of up to 83 current use pesticides (CUPs) in flowers (0.5 g sample weight) and 71 CUPs in pollen provision (0.1 g sample weight) via liquid chromatography-tandem mass spectrometry. Additionally, methods were developed for individual wild bees (*Osmia bicornis*), enabling the analysis of 65 CUPs in 0.02 g samples (females) and 45 CUPs in 0.01 g samples (males). The extractions used acidified acetonitrile (2.5% formic acid), with phase separation assisted by ammonium formate and clean-up via freeze-out. The validation showed limits of quantification between 0.00025 mg/kg and 0.05 mg/kg for flowers, 0.0002 mg/kg to 0.052 mg/kg for pollen provision, 0.0002 mg/kg to 0.08 mg/kg for female bees, and 0.00008 mg/kg to 0.1 mg/kg for male bees. The methods were applied to flowers, pollen provision, and post-pupal bees from agricultural sites. In total, 47 CUPs were detected in flowers, 35 in pollen provision, and 4 in post-pupal bees, with herbicides being most prevalent. This study highlights the exposure of pollinators to CUP mixtures, including emerging bees that have not yet been active in the environment. Our methods provide practical tools for monitoring CUP residues in small environmental samples, supporting the assessment of exposure in plant-insect matrices.

**Graphical Abstract:**

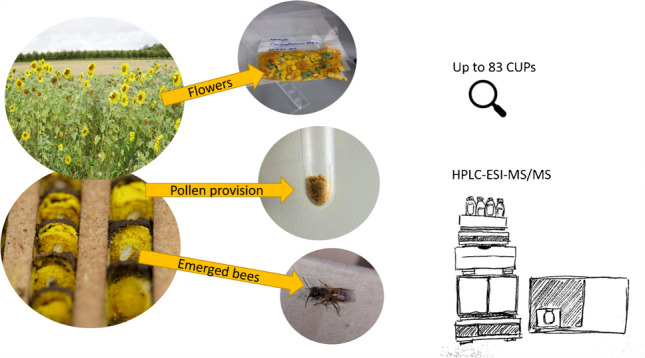

**Supplementary Information:**

The online version contains supplementary material available at 10.1007/s00216-025-05935-8.

## Introduction

The widespread use of synthetic pesticides for pest control has resulted in their ubiquitous presence in various parts of the biosphere [1, 2]. Current use pesticides (CUPs) have been detected in soil and vegetation at large scales, creating chemical landscapes and showing that CUPs are distributed further from the cropping area, as assumed by drift estimations [3, 4]. Exposure to pesticide mixtures has been suggested to be a major factor in the decline in pollinating insects [5, 6].

Recently, studies on insect exposure to pesticides in terrestrial ecosystems have increased, revealing multiple CUP residues in pollen from flowers [7, 8] or collected by honey bees (*Apis mellifera*, bee bread) [9–13] and bumble bees [8, 12]. CUP mixtures have also been recorded in [14–17] and on insects [18]. These findings emphasise the need for monitoring approaches to understand the presence of pesticide mixtures in terrestrial ecosystems and adjust current risk assessment practices [19].

Despite the direct contact of insects with flowers, research on CUP residues in blossoms remains limited. Existing approaches for the extraction of CUPs from flowers typically adapt protocols from food analysis (e.g. [20–22]). Sampling insects, flowers, or pollen is further constrained by the limited availability of matrix material in natural environments. Common extraction methods are based on the QuEChERS approach [23] and involve the use of a larger amount of sample material, usually 10 g for vegetables, fruits, and soil [24]. For pollen analysis, typically quantities between 1 and 5 g [25] are used. This is easily achievable in the context of honey bees, due to the storage of large quantities of pollen as bee bread. Obtaining sufficient wild bee pollen necessitates pooling larval pollen provision from multiple brood cells, obscuring individual exposure data. The development of small-scale extraction methods is essential to prevent the loss of information in combined samples, to evaluate the exposure of individuals separately [7], and to enable monitoring approaches.

We studied the red mason bee *Osmia bicornis*, which is also used as a surrogate test species in the registration process of pesticides [26]. The females of this species construct brood chambers in tubular passages in dead wood, with individual brood cells separated from each other by soil material. Each brood cell contains a supply of pollen, with the quantity stored varying depending on the sex of the developing larva. The pollen quantity ranges from 0.075 to 0.525 g of pollen, with 0.15 g being common [27]. Owing to the initial consumption of the pollen provision provided for the larvae, 0.1 g was chosen as a realistic sample amount for CUP analysis. With current approaches, it is not possible to analyse this low volume of pollen collected for the provision of single wild bee larvae.

For insects, in most published methods, multiple individuals are combined to obtain a sufficient sample size. Residues of CUP were recorded in honey bees *Apis mellifera* [10], as well as in wild bees [15], where multiple individuals were used to obtain between 0.083 g to 1.9 g [15] and 5 g of bees [10] for extractions. An exception is a British study, where CUPs from single bumble bees *Bombus terrestris* with an average weight of 0.098 ± 0.030 g were measured [7]. We aimed to determine the CUP residues in a single bee individual to compare them with the residues of stored pollen provision. Moreover, a small-scale method is more appropriate for monitoring approaches, as it results in less interference with the ecosystem. To the best of our knowledge, no methods for isolating single small solitary wild bees have been published thus far. Therefore, we had to develop a method that could be used for a single individual. The dry weight of a female *Osmia bicornis* varies between 0.022 g and 0.04 g, and for a male, it varies between 0.012 g and 0.024 g; therefore, 0.02 g and 0.01 g were selected to ensure the measurability of all the sampled individual females and males, respectively.

The central aim of this study was to develop efficient methods for the extraction and detection of multiple CUPs from low-mass samples of flowers (0.5 g), larval pollen provision (0.1 g), and newly hatched wild bee individuals (0.02 g and 0.01 g). The simplified extraction approach with minimal steps and components to reduce processing time, minimise the probability of human error, and, in particular, allows a cost-efficient processing of many environmental samples in a short time. The methods were validated and applied to field-collected samples of flowers, pollen provision, and newly hatched *Osmia bicornis.* Furthermore, we aimed to find a contamination path throughout the matrices by detecting the same pesticides in all the matrices.

## Material and methods

### Analytical standards, chemicals, and reagents

For the pesticide stock solutions, 93 CUP reference standards (purity ≥ 98%) were purchased either from Restek (Bellefonte, PA, USA) as multicomponent solutions (concentration, 100 mg/L) or from Dr. Ehrenstorfer (Augsburg, Germany) and Sigma-Aldrich (St. Louis, MO, USA) as single-component standards or solutions. A list of all standards is provided in Electronic Supplementary Material (ESM) Table S1. Single-stock and multicomponent solutions were combined into the final multicomponent solution (containing each analyte at a concentration of 10 mg/l) immediately before use to prevent the degradation of sensitive analytes. The final multicomponent solution was further diluted with HPLC-grade acetonitrile (≥99%, Honeywell, Seelze, Germany) to prepare five working solutions, each containing all the analytes at concentrations of 0.0005, 0.001, 0.01, 0.1, and 1 mg/l. All the solutions were stored at −20 °C in a freezer. For preparation of the HPLC mobile phases, LC-MS grade methanol (≥99.9%, Honeywell, Seelze, Germany), water (Merck, Darmstadt, Germany), buffer/additive, formic acid (≥99%, VWR International, Leuven, Belgium), and ammonium formate (NH_4_HCO_2_, ≥99%, Sigma-Aldrich, St Louis, MO, USA) were used. For the extraction, ammonium formate and HPLC-grade acetonitrile were used. Deuterium-labelled imidacloprid (purity ≥ 98.9%, Dr. Ehrenstorfer GmbH, Augsburg, Germany) was added to each sample prior to extraction as part of the quality assurance. For the clean-up steps, C18 (SPE Bulk Sorbent, Agilent Technologies, Santa Clara CA, USA), Z-Sep+ (Supel™ QuE Z-Sep+, Supelco®, Bellefonte, USA), and PSA (SPE Bulk Sorbent, Agilent Technologies, Santa Clara CA, USA) were used.

### Analysed pesticides

The 93 analysed current use pesticides (CUPs) were selected on the basis of records from the Julius-Kühn Institute (JKI) published in the PAPA database (JKI, 2019), as well as JKI records for the frequency of pesticides in 2016 and 2017. The most commonly used herbicides, fungicides, and insecticides for winter wheat, oilseed rape, maize, potato, and grapes were chosen. Additional information on the selected CUPs is available in a previous publication [28]. In contrast to the previously selected CUPs [28], avermectin (biocide) has been replaced by dimethoate (insecticide). The aim was to adapt the methods for flowers, pollen, and individual wild bees. The existing methods involve 93 target analytes, including 36 fungicides (F), 36 herbicides (H), and 21 insecticides (I). In this study, we report the determination of pure biologically active isomers, namely, dimethenamid-P (H), metolachlor-S (H), metalaxyl-M (F), spinosad-A (I), spinosad-D (I), and quizalofop-P (H), as the sum of isomers because commercial pesticide formulations often contain mixtures of active and nonactive stereoisomers.

### HPLC-ESI/MS-MS analysis

Analyte concentrations were measured via high-performance liquid chromatography coupled with tandem mass spectrometry via electrospray ionisation (HPLC-ESI-MS/MS; LC: Agilent Technologies LC 1260 Infinity II series, MS/MS: Agilent Technologies 6495 C, Santa Clara CA, USA). The mass spectrometer was operated in multiple reaction monitoring (MRM) mode with two characteristic MRM transitions per analyte (except for proquinazid, where only one MRM transition was used, ESM Table S2). Analytes were quantified via matrix-matched external calibration standards. For the calibration standards, blank matrix samples were fortified with working solutions to achieve final analyte concentrations of 0.025, 0.05, 0.1, 0.5, 1, 10, 50, and 100 µg/l. Calibration standards were injected before and after each sample batch or after every 20 samples to monitor for changes in the sensitivity of the mass spectrometer due to residues of the sample matrix in the ion source. Additionally, an internal standard was added to all the environmental samples prior to extraction to monitor for variation in the extraction procedure.

An Agilent MassHunter Workstation (Quant-my-way, Quantitative analysis for QQQ version 10, Agilent Technologies, Inc., Santa Clara CA, USA) was used to process the generated chromatograms. Analytes in the samples were identified by comparing the peak retention time and qualifier/quantifier MRM (ESM Table S2) ratios to those of the calibration standard at a similar concentration and comparing the values to the limits of detection (LODs, ESM Table S3) and quantification (LOQs, ESM Table S3) determined during method validation. The tolerance based on the qualifier/quantifier MRM ratios was defined to be within ± 20%.

### Samples and sample treatment

#### Materials used for method development

To prepare matrix-matched calibration series and perform recovery experiments of fortified samples, an uncontaminated reference material was sourced for each matrix. For this purpose, we collected flowers from the surroundings (parks, urban areas) of the Institute for Environmental Sciences Landau, RPTU, Germany (49°12′13.7″N 8°06′20.2″E). These flowers served as blank material but still contained residues of four CUPs (fluopyram, prosulfocarb, terbuthylazine and trifloxystobin). For the pollen method, commercially available pollen products, all of which contained CUPs, were tested. Alpine pollen (Blütenpollen Alpenmischung, GRAZE der Imkershop, Weinstadt, Germany) was chosen as the blank material because it contained the fewest CUPs (10 analytes). Adult red mason bees (*Osmia bicornis*, purchased from WAB-Mauerbienenzucht, Konstanz, Germany), which hatched in the laboratory, were used as blank material for the bee method. For the flower and pollen analyses, the concentrations of the CUP residue in the calibration series were adjusted by subtracting those detected in the blank material. The flowers and bees were freeze-dried (Alpha 1–4 L5 Cbasic, Martin Christ Gefriertrocknungsanlagen GmbH, Osterode am Harz, Germany) to ensure a homogeneous mixture. The drying process was not necessary for pollen provision, as pollen is a dry pellet that is easily homogenised during milling. All blank materials were stored in Eppendorf tubes at −20 °C prior to method development. The samples were milled to powder (pollen and flowers with an IKA, TubeMill control 100; IKA Staufen, Germany; bees with an oscillating mill, Typ MM 301, Retsch GmbH, Haan, Germany; 3 × 1 min; 30 × 1/s).

#### Environmental samples

All field samples were collected as part of the InsectExpo project [2]. The samples were collected at the same sites described in a previous work [2]. Nine agricultural sites (arable land (*n*=3), vegetable cultivation (*n*=3), and viticulture (*n*=3)) and adjacent meadows in the Rhineland-Palatinate region of southwestern Germany were investigated. Samples were taken in March and June 2021. Flower samples for this study were taken from meadows bordering the fields in the main wind direction to the east at distances of 1 m. After the flower species were identified, they were collected in separate sample bags (Rotilabo® sample bags, LDPE, Carl Roth GmbH + Co. KG, Karlsruhe, Germany). In the laboratory, all the samples were directly frozen (−20 °C). If enough flower material was available (more than 6 g dry weight), the flowers of an individual species were analysed. In several cases, flower mixtures had to be prepared to obtain the required amount of material (0.5 g), as the number of flower sources was limited. For mixtures, samples from the same date and sample site were pooled.

Nesting aids [29] and *Osmia bicornis* cocoons were placed in all the meadows (*n*=9) from the end of March to the end of July 2021. Initially, 13 female cocoons and 7 male cocoons were placed in paper rolls beneath the nesting aids at each sample site. After 2 weeks, 10 female and 10 male cocoons were added every 2 weeks to obtain constantly active bees, and pollen was continuously collected by the bees to provide pollen provision for the larvae. Nesting aids were built from three nesting boards of milled MDF boards (16 × 16 × 1.6 cm) with 10 nesting tunnels (8 mm, www.mauerbienen-shop.de, WAB-Mauerbienenzucht, Konstanz, Germany). To protect brood cells during handling, acetate sheets were fixed to each nesting board with adhesive strips. A 12-mm mesh net shielded the bees from predators, and nesting aids were secured with lashing straps on wooden posts, at least 1 m above the ground and 20 m from agricultural land, oriented south/southeast. The development of the nesting aids was regularly photographed to document brood cell establishment during the study. When a nesting board was full of brood cells or after 3 weeks, the nesting boards were taken to the laboratory before all the pollen was consumed by the larvae. Pollen samples were carefully taken from single brood cells via a spatula, and the larvae were subsequently fed purchased pollen but were not used for this study. Larval development from neighbouring cells, built within ± 1 day and at the same location, was observed, and after diapause (October to March, in a refrigerator, 3 °C), hatching success was documented. The newly hatched bees were directly frozen (−20°C). Bees that did not hatch after 4 weeks from the end of hibernation (14.03.22, first hatching bee: 20.03.22, last hatching bee: 08.04.22) were manually removed from the cocoon and frozen. All post-pupal bees (*n*=33) were later used for pesticide residue analyses.

### Development of the extraction procedure

For all the matrices, different extraction steps were investigated to achieve simple, quick, effective, and inexpensive extraction of multiple CUPs. The solvent extractions were based on extraction methods previously developed in our laboratory for soil and vegetation [28] and for biota [30]. For the experiments, a precise mass (Sartorius CP225D, Göttingen, Germany) of the sample was fortified with a standard solution of CUPs prepared in acetonitrile and evaporated to dryness under a fume hood (for at least 30 min). All shaking steps were performed via an end-over-end overhead rotator (Stuart STR4/2, Keison Products, Chelmsford, UK). For centrifugation, a MegaStar 1.6R (VWR, Radnor, USA) was used. Method average recoveries (recovery, REC %) and precision (relative standard deviation, RSD %) were evaluated by comparing fortified blank samples before extraction (*n*=3) and post-extraction fortified sample (*n*=1) for each matrix individually.

#### Flowers

For the pesticide extraction of flower material, 0.5 g dry weight (dw) of flower powder was weighed into a 50-ml CellStar® tube (PP conical, Greiner Bio-One, Frickenhausen, Germany). For each experiment, three samples were fortified with the working solution, while an additional three samples were left as blanks for spiking after the extraction process. For extraction, 10 ml of acetonitrile containing 2.5% formic acid (FA) and 0.25 g of ammonium formate was added to the fortified and blank flower samples. The samples were agitated for 1 h via an end-over-end overhead rotator and centrifuged (6 min, 3000 rpm). The supernatant was decanted and filtered through 0.2-μm filters (17-mm HPLC syringe filter, PTFE, hydrophobic, BGB Analytik, Lörrach, Germany) into 20-ml glass vials.

To eliminate potentially interfering substances, such as pigments or oils, we evaluated three dispersive solid-phase extraction (dSPE) clean-ups using either (1) PSA or (2) C18 as a sorbent or (3) freeze-out with cooled filtration. For dSPE, 1 ml of the supernatant was added to an Eppendorf tube containing 50 mg of sorbent, and the samples were then vortexed for 30 s and filtered into an HPLC vial. For freeze-out, the samples were subjected to freeze-out overnight at −20 °C (12 h), then placed on ice and filtered through PTFE filters (17 mm HPLC syringe filter, PTFE, pore size 0.2 µm; La-Pha-Pack, Langerwehe, Germany) into HPLC vials, ensuring that they remained cold during the filtration step. For the final method, 0.5 g dry weight of flowers sampled in the field was extracted with 10 ml of acetonitrile containing 2.5% FA and 0.25 g of ammonium formate, and a freeze-out was used as a clean-up.

#### Pollen provision

We used 0.1 g of pollen provision, as this was a realistic expected quantity per brood cell [27]. For the extraction of the pollen provision, 0.1 g fresh weight (fw) of pollen was weighed into 15-ml cellstar® tubes (PP conical, Greiner Bio-One, Frickenhausen, Germany). To remove proteins and fatty acids from the pollen matrix, different purification methods were tested. The supernatant was either (1) frozen overnight (freeze-out) followed by cooled filtration or cleaned by dSPE with (2) C18, (3) PSA, or (4) Z-Sep+. For the clean-up with PSA, 1 ml of H_2_O was added before agitation [31]. After solvent extraction, 1 ml of the supernatant was placed in a tube containing (1) 100 mg of C18, (2) 150 mg of MgSO4 + 25 mg of PSA, or (3) 100 mg of Z-Sep and vortexed for 30 s. The samples were centrifuged (6 min, 3000 rpm), and the supernatant was filtered on ice through 0.2-µm filters into an HPLC vial. For freeze-out, the supernatants of the samples were stored in a freezer (−20 °C, 12 h), after which the freeze-out samples were filtered before analysis. For the measurement of the field samples, the final method included the use of 2.5 ml of acetonitrile, 2.5% FA, 0.1 g of ammonium formate, and a freeze-out.

#### Post-pupal bees—*Osmia bicornis *

For individual red mason bee (*Osmia bicornis*) extraction, we tested an ultrasonic bath extraction method followed by purification via PSA, and Z-Sep and nitrogen evaporation. The method was partially based on the AOAC QuEChERS method [32] and a method that was previously established for small sample sizes of insects [30]. For extraction, 300 µl of water was added to 0.020 g of dried female bee powder in 2.5-ml Eppendorf tubes, which were subsequently vortexed for 30 s. Then, 2000 µl of 1% acetonitrile was added, and the mixture was vortexed again. Salts (10 mg of NaOAc, 40 mg of MgSO4, and 10 mg of NACl) were added, and the mixture was vortexed, followed by a 5-min ultrasonic bath (Sonorex Digitec, Bandelin, Berlin, Germany) and a centrifugation step (5 min, 4700 rpm). A total of 1800 µl of the supernatant was added to a vial containing Z-Sep+ (SupelTM QuE QuEChERS, Merck KGaA, Darmstadt, Germany) (0.0008 g per mg of bee powder) and then to a vial containing PSA (0.0008 g per mg of bee powder). The mixture was vortexed for 30 s and then centrifuged (5 min, 4700 rpm) again. The supernatant was evaporated to dryness under a nitrogen stream, and the resulting extract was dissolved in 500 µl of a solution containing water-methanol (H_2_O/MeOH, 70:30) and 0.1% FA. The solution was then filtered through PTFE filters (0.2 µm, 4-mm syringe filter, OTFE, Resteck, Bad Homburg, Germany).

Additionally, we tested a simple liquid extraction method, which has already been established for 93 CUPs for other environmental samples [28], with different clean-up steps ((1) C18, (2) PSA, (3) freeze-out + cooled filtration). For this method, 0.5 ml of acetonitrile, 2.5% FA, and 0.020 g of ammonium formate were added to 0.020 g of bee powder in a 2.5-ml Eppendorf tube, followed by 1 h of agitation, a centrifugation step (6 min, 3000 rpm) and the transfer of the supernatant by filtering into another Eppendorf tube. For three different clean-up steps, the supernatant was added to (1) C18 (0.0008 g per mg bee powder), (2) Z-Sep + PSA (0.0008 g per mg bee powder), or (3) freeze-out (−20 °C, 12 h) + cooled filtration. The samples were subsequently vortexed, centrifuged (5 min, 4700 rpm) and filtered (0.2 µm, 4 mm). For (3), the samples were centrifuged under cooled conditions (−10 °C).

For the post-pupal bees that hatched in the laboratory or were manually removed from the cocoons after consuming the provided pollen provision collected in the field, we used the final method of 0.5 ml of acetonitrile containing 2.5% FA, 0.02 g of ammonium formate, and a freeze-out as a clean-up for 0.02 g of dried females. The method was also validated for 0.01 g of dried males with 0.5 ml acetonitrile 2.5% FA and 0.01 g of ammonium formate; after agitation and centrifugation, samples with 0.01 g matrix were evaporated for 30 min using nitrogen. The samples were resolved in 0.25 ml acetonitrile 2.5% FA and further treated like the 0.02 g samples.

### Method validation

The optimised extraction methods were validated for the analysis of 93 CUPs in flowers, pollen, and female and male bees by analysing accuracy (recovery, REC (%)), precision (relative standard deviation, RSD (%)), linearity (*R*^2^), matrix effects (ME (%)), and limits of detection and quantification (LODs and LOQs). If one of the validation parameters (REC, RSD, or ME) was not achieved, the substance failed to be validated.

RECs were determined by calculating the ratio of the response of blank samples fortified with two concentrations prior to extraction to the response of the blank samples fortified after extraction with a minimum of three repetitions. The REC needed to be in the range of 70–120% [33]. The RSDs of the methods were evaluated by determining the relative standard deviations (RSD=(100*standard deviation)/average) of at least three repeated extractions of each matrix spiked at two concentrations (for flowers, pollen provision, and female bees (20 mg): 0.01 mg/kg and 0.1 mg/kg dry weigh; for male bees (10 mg): 0.1 mg/kg and 0.25 mg/kg). Acceptable precision was achieved if the RSD was <20% [33]. *R*^2^ was evaluated via matrix-matched calibration curves prepared as described above and in a concentration range of 0.00025–2 mg/kg (8 concentrations). Linearity was considered acceptable if *R*^2^>0.99. The ME was evaluated by comparisons of the slopes of the calibration curves (eight concentrations) in solvent only (acetonitrile, 100%) and in the matrix. The percent increase or decrease in the slope of the matrix-matched calibration curve was measured in relation to the solvent-only curve. MEs less than 20% were considered acceptable [33]. The LODs and LOQs were determined from spike samples (*n*=5 for each of the eight calibration levels). The LOD (LOD = (3* standard derivation of the response)/slope of the calibration curve) was determined as the minimum amount of analyte for the quantifier multiple reaction monitoring (MRM) transition detected with a signal-to-noise (*S*/*N*) ratio of three, and the LOQ (LOQ= (10*standard derivation of the response)/slope of the calibration curve) was determined as the minimum amount of analyte detected at an *S*/*N* ratio of ten. For the determination of the LOD and LOQ, five blank samples per concentration were fortified after extraction. The signal-to-noise ratio was calculated with a root mean square (RMS) algorithm in the MassHunter Workstation.

### Statistical analysis

For significant differences between the methods, an ANOVA test (package: stats [34]) and a post hoc *t*-test were conducted [35]. Plots were produced with ggplot (package: ggplot2 [36]). For the environmental samples whose detection results were below the limit of quantification but above the limit of detection, the detections were not used for cumulative mixture CUP concentrations but were counted for the number of CUPs. Owing to nonnormally distributed data, we used the Kruskal-Wallis test (package: stats [34]) and Dunn post hoc test (package: FSA [37]) for multiple comparisons between types of agricultural sites (arable sites, vegetable sites, viticulture sites) per matrix and between sample types (flowers, pollen provision, post-pupal bees). For cumulative concentrations, the data were log transformed. All the statistical analyses were performed via R software (version 2023.06.2+561, R Core Team, 2022).

## Results and discussion

### Method development and validation

To detect multiple CUPs in environmental samples such as flowers, pollen provision, and wild bees, multiresidue methods capable of analysing a broad range of analytes have been developed. To ensure appropriate extraction conditions for acidic and basic compounds, acetonitrile with formic acid and ammonium formate were used as buffers [38]. The aim was to develop effective and inexpensive methods that enable a large number of environmental samples to be analysed quickly.

#### Flowers

**Fig. 1:**
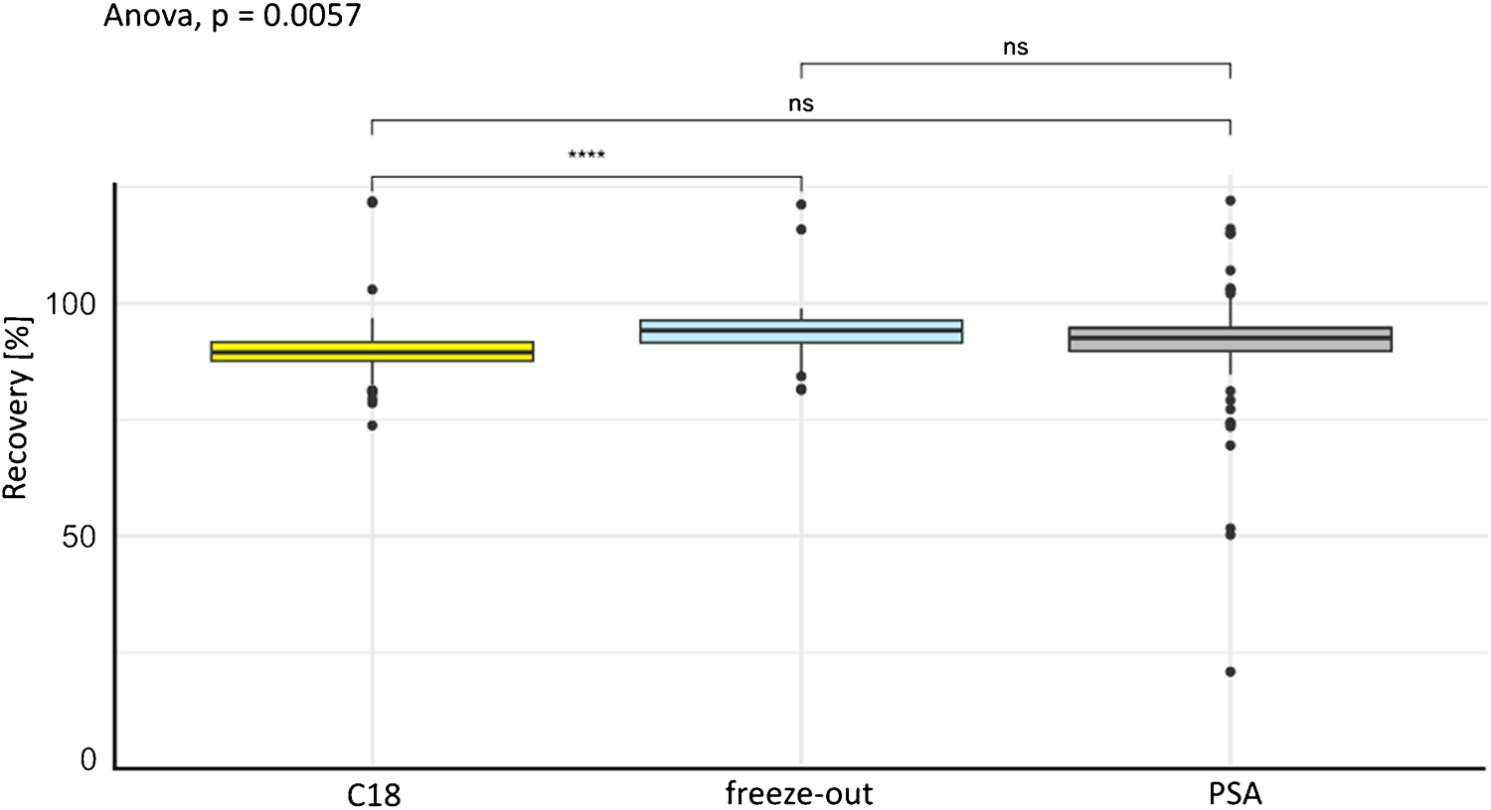
Boxplot of recoveries [%] over all substances (*n*=93 CUPs) with the tested methods (*n*=3, dSPE clean-up with C18 (yellow) and PSA (grey), as well as a freeze-out (light blue) as a clean-up) for 0.5 g of flower material. Lines indicate significance (****) or non-significance (ns) (*t* test, CL= 95%)

For the flower extraction method, different clean-up methods were tested: overnight freeze-out and dSPE with C18 and PSA (see Fig. 1). The average REC after dSPE with C18 (REC: 90 ± 6%) was statistically significantly lower than that after the freeze-out method (freeze-out: 94 ± 5%; see Fig. 1). No statistically significant differences were found between the PSA method (PSA: 91 ± 12%) and the freeze-out or the PSA and C18 methods. PSA and C18 are commonly used for clean-up steps. PSA is capable of retaining fatty acids from acetonitrile [39]. C18, on the other hand, is characterised by non-polar properties and is able to adsorb fatty acids due to hydrophobic interactions. Due to the low solubility of lipids and proteins, these compounds precipitate on the tube surface during a freeze-out process. As a result, the supernatant can be transferred without these compounds. Although PSA showed comparable recoveries, freeze-out was universally favoured due to its simplicity, lower cost, and lower methodological complexity and as the analytical sensitivity was sufficient for multiple target analytes and an additional clean-up step would have significantly reduced throughput. Owing to the simplicity of the method, overnight freeze-out was chosen over PSA.

##### **Validation**

Among the 93 CUPs, 83 achieved the aforementioned validation criteria. The RSDs were within the acceptable range for 88 and 92 of the 93 tested CUPs, depending on the fortification level. Depending on the fortification levels, RSD ranged from 1.3% (hexythiazox, I) to 12.7% (difenconazole, F) for validated CUPs in the flower samples, with 98% (*n*=81 of 83 validated CUPs) of the CUPs having RSDs smaller than 10%. Acceptable REC were achieved for 87 and 91 of the 93 CUPs depending on the fortification level, with average recoveries ranging between 76.3% (propamocarb, F) and 118.9% (flonicamid, I). The *R*^2^ was ≥ 0.99 for 92 of the 93 tested CUPs. For the validation of the CUPs in flowers, 90 CUPs had MEs (between −20 (clomazone, I) and 13% (benalaxyl, f)) considered negligible (ESM Table S3).

The LOQ for flowers varied from 0.00025 mg/kg (propaquizafop, H) to 0.02 mg/kg (aminopyralid, H) (ESM Table S3). Among the 83 CUPs, 20 had LOQs ranging from 0.00025 mg/kg to 0.001 mg/kg, 49 had LOQs between 0.0011 mg/kg and 0.01 mg/kg, and 5 had LOQs between 0.010 mg/kg and 0.1 mg/kg (ESM Table S3). To further improve the LOQs, additional experiments with different extraction steps could be conducted. For example, evaporating the solvent and reabsorbing it into another solvent could improve recovery.

The detection limits of our method are lower than those of other studies investigating CUP residues in flowers (LOD: 0.006 mg/kg for LC analytes and 0.016 mg/kg for GC analytes, LOQs not mentioned [15] and LOQs: <0.01 mg/kg, LODs not mentioned [20]). In contrast to our method, the methods used in these studies were not initially developed and validated for flowers. Low LOQ values are particularly important when analysing environmental samples, as CUPs are often present at low concentration levels and in complex mixtures (e.g. (2), see also the “Method application” section). Even trace-level CUP residues on flowers can lead to contact exposure for flower-visiting insects. Although the effect of such low concentration exposure has not yet been studied, this exposure pathway should be further investigated [6]. For example, acetamiprid, a common insecticide, has a contact lethal dose (LD50) of 9.26 µg/honey bee [40]. While it seems unlikely that this dose is reached through contact with flowers, it is important to note that acetamiprid present in mixtures can exhibit synergistic effects on honey bees [41]. Moreover, lethality threshold concentrations provide only a rough estimate of toxicity. The distinction between lethal and sublethal effects is often difficult and depends on various factors, including individual fitness or population health and environmental factors [42]. In three Belgian studies, 10 g of cut flowers was analysed to identify approximately 500 target CUPs [20, 22]. The samples were analysed by a laboratory specialising in residue analysis for food and herbs, following a QuEChERS approach [43] involving the use of 10 ml acetonitrile with 1% acetic acid and QuEChERS salts (4 g anhydrous magnesium sulfate, 1 g sodium acetate), shaking and a sonication step in an ultrasonic bath [22], and the use of clean-up tubes [20, 22]. The extracts were measured both by liquid chromatography (LC) and gas chromatography (GC). This enables the detection of up to 500 CUPs. However, the use of magnesium sulfate leads to an exothermic reaction, and this increase in temperature worsens the detection of more unstable pesticides [44]. Moreover, ultrasonic baths have also been used to remove CUPs from eatables [45]. Ultrasonic sonification also occurs at relatively high temperatures (e.g. between 26 and 36 °C [46]), leading to loess with temperature-unstable CUPs. Comparisons of the recovery of CUPs extracted with ultrasonic bath or milder conditions due to overhead shaking obtained better results for the selected CUPs [28]. Furthermore, a sample amount of 10 g is achievable in the context of commercial flowers but not in natural habitats. In this context, floral pollen samples were analysed; however, flower-visiting insects are exposed also via the flower surface, which serves as a contact exposure matrix for these insects. This is particularly of concern regarding CUPs with high lipophilicity, as these substances might permeate the cuticular lipid layers of insects during foraging or nesting [47].

In order to determine the exposure of wild bees and honey bees to CUPs from flowers from field margins of agricultural land, in a Californian study, flowers were extracted by a pressurised liquid extraction followed by a solid-phase extraction clean-up and analysed for 163 CUPs [17]. Here, a 0.3-g sample was used, which is similar to our method (0.5 g of flower). However, the extraction involves temperatures up to 100 °C [15], causing possible losses, as discussed above. The samples were again measured by both LC and GC, which cover a broader range of CUPs. An additional measurement of our extracts with GC-MS/MS would be a possibility to cover more CUPs. Owing to the absence of water in our extracts, an adaptation of the method for additional measurements with GC is imaginable.

#### Pollen provision of bee larvae

**Fig. 2:**
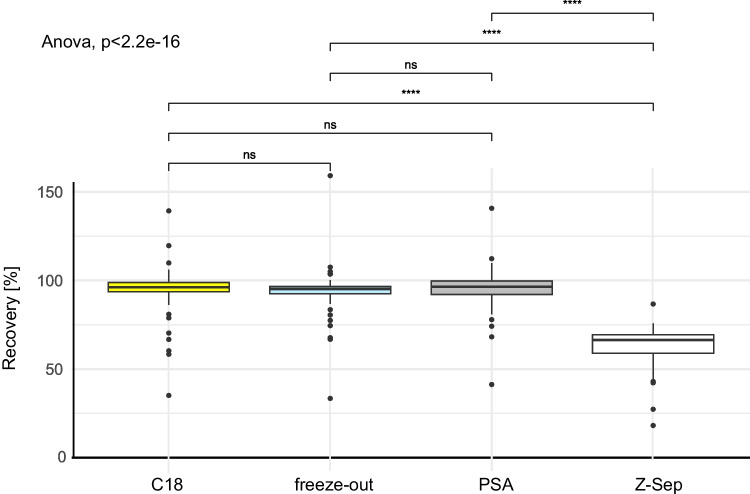
Boxplot of recoveries [%] over all substances (*n*=93 CUPs) with the tested methods (*n*=4, dSPE clean-up with C18 (yellow), PSA (grey), and Z-Sep (white), as well as a freeze-out (light blue) as a clean-up) for 0.1 g pollen provision. Lines indicate significance (****) or non-significance (ns) (*t* test, CL= 95%)

Common clean-up approaches for pollen samples involve manual dSPE with C18, PSA (e.g. [11]), or Z-Sep as sorbents (e.g. [48]). Z-Sep is a mixture of C18 and zirconium dioxide sorbents and is used to reduce fats and proteins; it was successfully used in other studies investigating extraction methods for pollen [49]. However, in our experiments, the REC achieved via Z-Sep was generally below the desired range for most of the analytes. This outcome (mean REC: 64 ± 10% for all 93 CUPs) suggests that the quantity of Z-Sep might have been excessive and that further optimisation with reduced amounts could yield more recoveries within the range. In contrast, the recoveries for most of the analytes fell within the target range of 70–120% when clean-up methods with C18 (mean REC: 95 ± 11% for all 93 CUPs), PSA (mean REC: 99 ± 34% for all 93 CUPs), or freeze-out (mean REC: 94 ± 11% for all 93 CUPs) were used. No statistically significant differences were detected between PSA, C18, and freeze-out (see Fig. 2, ANOVA, *p* value = 2.2e^−16^, ESM Table S3). The method with overnight freeze-out was chosen because of its lower cost and fewer steps, as it requires no sorbent and is thus simpler, reducing the likelihood of human error. However, the use of further clean-up reagents might decrease MEs for some substances, as has been shown in other studies (e.g. [49, 50]).

In another method, 0.1 g of pollen material collected directly from flowers was used, as the authors aimed to analyse the exposure of pollen from individual plant species [7]. This method would have also been suitable for our sample amount of 0.1 g. However, the extraction process involves several steps, including dSPE, evaporation under vacuum, and reconstitution, increasing the time and complexity of the procedure.

Freeze-out was previously used in other pollen or bee bread extraction methods. For the analyses of 267 different CUPs in bee bread, a freeze-out alone did not yield satisfying results; therefore, two dSPE steps were included [38]. Other authors reported that an additional dSPE with PSA+C18 followed by an SPE step with Z-Sep after freeze-out was most efficient for the extraction of 220 CUPs with LC and GC [49].

##### **Validation**

The method was validated successfully for 71 of the 93 tested CUPs (ESM Table S3). RSDs were in acceptable ranges for 83 and 90 of the 93 tested CUPs depending on the fortification level, ranging between 2.5% (picoxystrobin, F) and 17.9% (boscalid, F). Acceptable recoveries were achieved for 87 and 93 of the 93 tested CUPs again depending on the fortification level, with REC between 88.2% (myclobutanil, F) and 119.4% (cymoxanil, F). Linearity showed acceptable coefficients (*R*^2^ ≥ 0.99) for 92 of the tested CUPs. The MEs were negligible for 80 CUPs; MEs for validated CUPs (*n*=71) ranged from −18.8% (acetamiprid, I) to +17.0% (florasulam, H).

The LOQs ranged from 0.0002 mg/kg (propamocarb, F) to 0.052 mg/kg (bromoxynil, H) (ESM Table S3). Among all the analysed CUPs in pollen provision, 20 had LOQs lower than 0.001 mg/kg, 46 had LOQs between 0.0011 mg/kg and 0.01 mg/kg, and 5 had LOQs between 0.011 mg/kg and 0.1 mg/kg. A recent study has shown that environmental low concentration of CUPs in mixtures leads to sublethal effects on insects [51]; for this reason, it was desired to establish low LOQ values. The overnight freeze-out method was finally validated for 71 CUPs.

#### Post-pupal bees

For post-pupal bees, we compared the method that we used for pollen provision extraction to solid-liquid extraction via acidified acetonitrile followed by dSPE clean-up via Z-Sep and PSA, which has been applied to extract multiple analytes from small arthropod samples by other authors (“biota” method) [30].

**Fig. 3:**
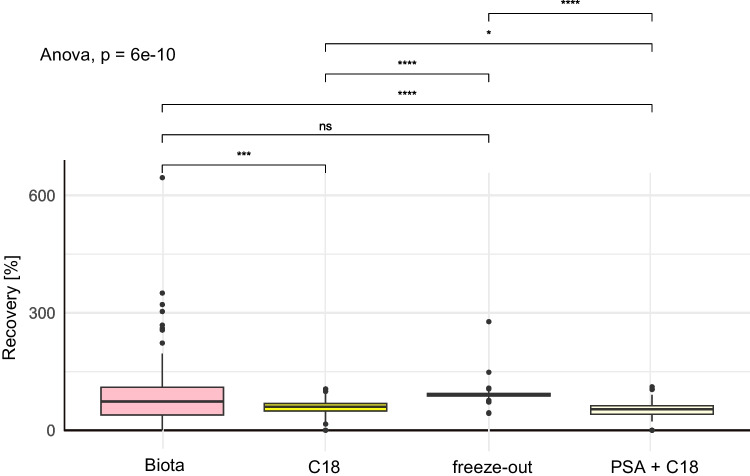
Boxplot of recoveries [%] over all substances (*n*=93 CUPs) with the tested methods (*n*=4: the “Biota” extraction method (red) based on an extraction method for biota [52], extractions based on the proposed pollen provision extraction and a dSPE clean-up with C18 (yellow) and PSA+C18 (eggshell) as well as a freeze-out (light blue) as a clean-up) for 0.02 g bee powder. Lines indicate significance (*, ***, ****) or non-significance (ns) (*t* test, CL= 95%)

The REC was statistically significantly lower (ANOVA, *p* < 0.05) across all the tested CUPs for the dSPE with C18 (mean REC: 57 ± 20%) and PSA with C18 (mean REC: 50 ± 26%) than for the freeze-out samples (mean REC: 92 ± 23%). The mean RECs for the “biota” method were 94 ± 94%, which was significantly different (ANOVA, *p* < 0.05; ESM Table S8) from those of the methods including dSPE (see Fig. 3). We opted for the pollen provision-based method with a freeze-out of our environmental samples, as RECs were within the range for more CUPs and the simplicity of this method, as discussed previously for the other matrices.

##### **Validation**

The methods were successfully validated for 65 (female bees, 20 mg dw) and 45 (male bees, 10 mg dw) CUPs. Validation for female bees failed due to poor REC values (*n*=21, ESM Table S3), poor RSD (*n*=16), or poor ME (*n*=5). For the male matrix, the most common unfulfilled criterion was the ME (*n*=41, ESM Table S3). In contrast to the other matrixes, extracts of male bees were evaporated to dryness before resolving in acetonitrile, whereby unremoved fats or other co-extracts can be concentrated, which might have led to higher ME. Additional steps with hexane could be tried out in further investigations, as a fraction of hexane in acetonitrile is known to remove lipids from extracts [14].

RSD were in an acceptable range for 77 and 83 of the 93 CUPs, depending on the fortification level, with acceptable RSDs between 3.5% (sulfoxaflor, I) and 19.9% (tebufenozide, I) in the female bee samples, with 28% being below 10% (ESM Table S3). The RSD for males (10 mg) was acceptable for 87 and 89 CUPs, depending on the fortification level; RSDs within the acceptable range were between 0.09% (bentazone, H) and 17.5% (bentazone, H), with 59% being below 10% (ESM Table S3). RECs were acceptable for 72 and 89 of the 93 tested CUPs in females, with acceptable RECs ranging from 76.4% (bromoxynil, H) to 119.5% (bixafen, F). In male bee matrix, 82 and 88 CUPs fulfilled the REC criteria, RECs for acceptable CUPs ranged from 71.3% (flupyradifurone, I) to 93.8% (propamocarb, F). The linearity showed acceptable coefficients for all CUPs (*R*^2^ ≥ 0.99) in females and for 89 CUPs in males (ESM Table S3). MEs were negligible for 88 (16.5% (difenoconazole, F) to 13.1% (propamocarb, F)) of 93 CUPs (ESM Table S3) in females and for 52 CUPs (−19.9% (metamitron, H) to 19.3% (bentazone, H)) in males. The LOQs ranged from 0.0002 mg/kg (propamocarb, F) to 0.08 mg/kg (pymetrozine, I) (ESM Table S3) for females and from 0.003 mg/kg (fenpropimorph, F) to 0.1 mg/kg (sulfoxaflor, I) for males. In single female bees, for 5 CUPs, the LOQs were lower than 0.001 mg/kg, 51 ranged between 0.001 mg/kg and 0.01 mg/kg, and 9 had LOQs between 0.011 mg/kg and 0.10 mg/kg. For males, 30 CUPs had LOQs between 0.001 mg/kg and 0.01 mg/kg, 14 CUPs had LOQs between 0.01 mg/kg and 0.1 mg/kg, and sulfoxaflor had LOQs above 0.1 mg/kg (ESM Table S3). Compared to our other methods, LOQs were higher in male post-pupal bees, which is probably caused by the low sample volume, which has an influence on LOQ values [33]. Lower LOQ values would be better to cover also in lower traces of CUPs present in the insects; however, our methods give first insights into the presence of CUPs in post-pupal single individuals.

On the basis of the preselection of pesticides in 2019 [28], 14 out of the 25 substances with a sales volume exceeding 100 tons in Germany [53] are also included in the validated extraction method for male bees, although we cover the fewest CUPs compared with pollen provision and flowers with this method (ESM Table S3). Our method is a quick and easy approach for detecting multiple CUP residues in individual wild bees.

#### Limitations—all three matrices

We started this method development with a wide range of CUPs; however, after validation, only a subset of all these CUPs were covered. The preselection of relevant compounds was conducted in 2019 [28], and method development started in 2021. Owing to the time shift and the ongoing revision of CUP approvals, some CUPs included in the methods are no longer approved for use in Germany (ESM Table S11) [54]. Continuously updating the method for approved CUPs is necessary to cover relevant or best-selling CUPs. Our methods cover a wide range of approved CUPs; moreover, CUPs that have recently been banned might also be detected in environmental samples. It has already been shown that pesticides are detected in environmental samples beyond their use [2, 55]. Another approach would be to adapt the proposed methods for further CUPs via GC analyses. MEs have also been observed for certain compounds; therefore, further investigations of the reduction in these effects would be interesting. Although we found similar or better recoveries with the freeze-out instead of C18 or PSA, other agents could be useful to further reduce ME. MEs possibly also lead to observed incongruence in the reported LOQ values within a matrix between the compounds as well as between the matrices per compound, where also the varying sample amount has an influence.

### Method application

As a proof of concept, the validated methods were applied to different environmental samples of flowers, pollen provision collected from the field, and hatching bees.

#### CUPs in post-pupal bees, pollen provision, and flowers

The different sample types had significantly different average numbers of CUPs per sample (ANOVA, Tukey’s *p* < 0.05, ESM Table S10), with all being significantly different from the other sample types (ANOVA, Tukey’s *p* < 0.05, ESM Table S10). This finding is in line with the results of a study in Michigan, USA, in which farmed blueberry flowers, honey bees, pollen collected from honey and bumble bees, and wax were sampled [56]. However, they detected more CUPs per sample than we did, possibly due to their screening for 261 CUPs and the analysis of a larger number of samples.

Across all the environmental samples, three CUPs (clomazone (H), metrafenone (F), and pendimethalin (H)) were detected in all three matrices, 25 in two of three, and 15 in a single matrix (ESM Table S7). The samples from all three matrices within the sample site and time period contained various CUP residues (ESM Table S4). Only residues of clomazone (H) and pendimethalin (H) were detected in all three matrices from the same time and site (Table 2; ESM Table S4). In a vegetable field (O3) at the beginning of June, clomazone was detected at a concentration of 0.002 mg/kg dw in flowers, 0.002 mg/kg fw in pollen provision, and 0.262 mg/kg in hatched bees (Table 1). Additionally, pendimethalin was recorded at a vegetable site in late May at 0.353 mg/kg dw in flowers, while the levels in pollen provision and post-pupal bees were below the LOQ (Table 1). Another pendimethalin detection occurred at a different vegetable site (O3) at the beginning of June, with 0.138 mg/kg dw in flowers and 0.126 mg/kg fw in pollen provision; no female bee was extracted for which reason no pendimethalin could have been detected (Table 1), as the method validation of pendimethalin failed for male bees. Pendimethalin and clomazone can be applied in vegetable cultivation (e.g. [57]). Matching detections in flowers, pollen provision, and post-pupal bees support the hypotheses of an exposure path over the crops to adjacent plants. However, contrary to expectations, most of the CUPs detected did not match the matrices, indicating that several pesticide routes can be used to contaminate the environment. The pollen provision analysed and the provision consumed by the larva may differ, even if the brood cells were built on the same day. This is particularly likely since bees also use mixtures of different pollen types with different exposure levels depending on landscape heterogeneity and flower diversity. Owing to the high variability in pollen provision, it is possible that the larval provision analysed for CUPs contained different residues than the pollen consumed by the larva. This is also in line with discrepancies between the detection of CUPs in pollen provision and flowers (ESM Table S7). Flowers were sampled right next to the field, while the bees possibly collected their pollen supply from other plants. To verify a floral uptake, a laboratory experiment would be necessary to evaluate how CUP residues applied on plants or next to flower plants are taken up to the flowers, in the pollen and exposed larvae due to pollen provision.

**Table 1:** Concentrations of clomazone (H) and pendimethalin (H) from samples taken at the same site (O1, O3) and during the same time period (late May (23.05–26.05.21) or starting in June (01.06–04.06.21)) in flowers (mg/kg dw), pollen provision (mg/kg fw), and post-pupal bees (mg/kg dw). “n.d.” indicates no detection

Matrix	*n*	Type of agricultural site	Site	Time period	Clomazone (mg/kg)	Pendimethalin (mg/kg)
Flower	1	Vegetables	O3	Start June	0.002	0.138
Pollen provision	1	Vegetables	O3	Start June	0.002	0.126
Post-pupal bee	1	Vegetables	O3	Start June	0.262	No female
Flower	1	Vegetables	O1	Late May	0.006	0.353
Pollen provision	2	Vegetables	O1	Late May	n.d.	LOQ
Post-pupal bee	1	Vegetables	O1	Late May	n.d.	LOQ

The concentrations (Table 2) were significantly different between the sample types (ANOVA, Tukey’s *p* < 0.05, ESM Table S10). The maximal observed concentration in pollen provision was detected for the fungicide azoxystrobin (3.28 mg/kg pollen provision, ESM Table S4); contrary to expectations, azoxystrobin was not detected in any of the newly hatched bees (ESM Table S4). There are currently no available data on the degradation times of pesticides in pollen. Within the time of exposed larvae (May/June 2021) and post-pupal bee (March 2022), CUPs might have degraded in the bees. More importantly, CUPs can be degraded and detoxified in bees via metabolic processes [58], and microbes in the insect gut play a major role in detoxification processes [59]. Analysing the cocoon or feces might give insights if the measured CUPs in pollen provision were stored during detoxification processes in the cocoon or feces. Moreover, CUPs are transported by insects through different life stages; higher concentrations were measured in midges larvae exposed to CUPs than in adults after emergence [52]. The lower concentrations in adult midges could be explained by the excretion of contaminants during the pupation process [52], which could also explain the observed low number of contaminants detected in post-pupal bees.

**Table 2:** Summary table of CUP residue detection from flowers, pollen provisions collected from *Osmia bicornis*, and post-pupal single wild bees (*Osmia bicornis*) sampled in Rhineland-Palatinate in May and June 2021. Means, standard errors (SE), medians, and maximum (Max) for the numbers of CUPs detected per sample for each sample and types of agricultural sites and cumulative concentration (mg/kg) per sample for each sample type (flowers, pollen provision, post -pupal bees) and type of agriculture site (arable crops, vegetables, viticulture)

	Number of CUPs per sample					Cum. Conc. (mg/kg) per sample			
	Mean	SE	Median		Max	Mean	SE	Median	Max
*Flowers*									
Arable crops (*n*=7)	10.90	3.24		12	15	2.311	5.260	0.245	14.218
Vegetables (*n*=8)	11.40	2.97		11.5	15	0.394	0.487	0.162	1.199
Viticulture (*n*=5)	5.60	3.36		5	11	0.395	0.836	0.0209	1.890
*Pollen provision*									
Arable crops (*n*=4)	1.25	0.50		1	2	0.005	0.007	0.001	0.016
Vegetables (*n*=12)	7.33	4.36		8	15	0.393	1.170	0.047	4.101
Viticulture (*n*=19)	4.79	3.17		4	14	0.161	0.503	0.019	2.214
*Post-pupal bees (O. bicornis)*									
Arable crops (*n*=5)	0.40	0.55		0	1	0.005	0.01	0	0.022
Vegetables (*n*=11)	0.55	0.52		1	1	0.03	0.079	0	0.262
Viticulture (*n*=17)	0.24	0.44		0	1	0.001	0.006	0	0.023

#### Flowers

Among the 83 target CUPs, 35 (21 fungicides, 12 herbicides, and 2 insecticides) were recorded in the flower samples (ESM Table S4). CUP residues were detected in all the flower samples (100%, *n*=20). On average, 11 CUPs were recorded in flowers from arable and vegetable sites (min: 8 vegetable and 5 arable sites; max: both 15). However, average numbers were statistically greater only in vegetable sites than in flowers from viticulture sites, with an average of 6 CUPs (min: 2, max: 11; Table 2; Kruskal-Wallis test, *p* < 0.05; ESM Table S9). In a Californian study, flowers from a field border were sampled [17]; the exact number of CUPs per sample was not given, but from graphs, it can be estimated that, on average, approximately 3 CUPs were detected per flower on the basis of an analysis of 168 pesticides. The Californian study measured pollen- and nectar-producing structures isolated from flowers [17], whereas we measured the whole flower, assuming not only exposure from these structures but also exposure from direct contact, e.g. during nectar consumption. This discrepancy might explain the differences in the detected numbers.

In our samples, no statistically significant differences were measured between the cumulative concentration in flower sample from the different sites; it was 2.311 mg/kg dw in flowers from arable sites and 0.394 mg/kg dw and 0.395 mg/kg dw in flowers from vegetable and viticulture sites (Table 2, Kruskal-Wallis test, *p* > 0.05; ESM Table S9).

The presented methods for CUP extraction method developed allow the systematic study of CUPs in naturally occurring flowers, and, e.g., in flower strips, which were created to promote biodiversity. However, our results show that it is more likely that such flower strips can be problematic for insects due to the presence of CUPs.

#### Pollen provision of bee larvae

Overall, 35 CUPs out of our 71 CUPs included in our target method were detected: 21 fungicides, 10 herbicides, and 4 insecticides. In 94% of all collected *O. bicornis* pollen provision samples (*n*=33 of 35, ESM Table S4), CUP residues were detected. A significantly lower average number of CUPs was detected in pollen provision from arable sites (mean: 1.25, min: 1, max: 2, Table 2) than in pollen provision from vegetable sites (mean: 7.33, min: 0, max: 15, Table 2; Kruskal-Wallis test, *p* < 0.05, ESM Table S9). No significant differences were detected between pollen provision from viticulture sites (mean: 4.79, min: 0, max: 14, Table 2) and arable or vegetable sites (Kruskal-Wallis test, *p* > 0.05; ESM Table S9). One possible reason for this difference could be the varying management practices between arable and vegetable fields. In arable fields, crops remain on the field for longer periods of time. However, vegetable fields have greater turnover of crops during the season, which also results in more pesticide applications.

The number of CUPs in daily pollen samples from agricultural sites (average across sites: 5) aligns with daily samples from honey bee collected pollen in a fruit-growing area ([11]: average 5 CUPs). In contrast, more CUPs were detected in mixed pollen provisions collected by *Osmia bicornis* at urban sites (mean of 13 CUPs) and in orchards (mean of 17 CUPs) in the Czech Republic, where pollen provision was pooled over 14 to 23 days and 96 pesticides and their metabolites were analysed [60]. These methodological differences, along with the number of target analytes, may explain the variation in CUPs per sample. However, it is evident that CUP mixtures are present in pollen provision. Typically, CUPs are tested individually for their effects on nontarget organisms; however, research has demonstrated that *Drosophila* larvae chronically exposed to CUP mixtures (9 CUPs, no insecticide) at field-realistic concentrations exhibit widespread sublethal effects on larval development, behaviour, and reproduction [51]. It is therefore likely that the CUP mixtures detected here also have an effect on larvae even at low concentrations.

Significant differences were detected for the mean cumulative concentrations of pollen provision from vegetable sites (mean: 0.393 mg/kg dw, Table 2), which were significantly greater than those from arable sites (mean: 0.005 mg/kg dw, Table 2; Kruskal-Wallis test, *p* value < 0.05, ESM Table S9), and no further statistically significant differences were identified.

#### Post-pupal bees

Among the 65 CUPs in female solitary bees and 45 CUPs in male solitary bees, four were detected in post-pupal bees (clomazone (H, *n*=4), metrafenone (F, *n*=1), pendimethalin (H, *n*=6), and propamocarb (F, *n*=1)). On average, 0.4 CUPs were detected in post-pupal bees from arable sites, compared with 0.6 CUPs per post-pupal bees from vegetable sites and 0.2 CUPs per post-pupal bees from viticulture sites (Table 2). Overall, 14 of the 33 *Osmia bicornis* (42.4%) successfully hatched. All remaining bees (*n*=19) were manually removed, as it was assumed that they may have died due to pesticide exposure. CUP residues were detected in 10 of the 19 manually removed bees and in 3 of the 14 successfully hatched bees (39.4%, *n*=13 of 33; ESM Table S4). However, within the post-pupal bee samples, no significant differences were detected in the cumulative concentration or the number of CUPs per sample between the hatches and manually removed bees (Wilcoxon rank-sum test, *p* value > 0.05; ESM Table 9).

The highest concentrations (mean: 0.03 mg/kg, maximum: 0.262 mg/kg, Table 2) of CUPs were measured in bees from the vegetable sites compared with those from the arable sites (mean: 0.005 mg/kg, maximum: 0.022 mg/kg, Table 2) and viticulture sites (mean: 0.001 mg/kg, maximum: 0.023 mg/kg, Table 2); however, these observed differences were not statistically significant (Kruskal-Wallis test, *p* > 0.05, ESM Table S9). Other studies have investigated the effects of CUP residues on insects caught in the field [15, 16] and detected several CUPs, which is not surprising because these insects fly in an agricultural environment. In addition, CUPs have been detected in mixed samples of newly hatched adult aquatic insect CUPs in natural wetlands [30]. Moreover, the authors reported bioaccumulation through the food web [30]. This bioaccumulation due to the CUP load on insects is also considered a reason for CUP detection in bat guano [61].

To the best of our knowledge, this is the first study investigating CUP residues in freshly post-pupal bees, which were not exposed to CUPs in an experimental setup; for this reason, the number of CUPs cannot be compared with other data. Here, we report that newly developed insects under environmental conditions also contain CUP residues. These results indicate the widespread and lifelong exposure of insects to CUPs and the potential for bioaccumulation through the food web.

## Conclusion

In this work, analytical methods for the parallel quantification of 83 CUPs in flowers, 71 CUPs in pollen provision, and up to 65 in female bees (0.02 g, in male bees (0.01 g) 43 CUPs) were developed and validated. The developed methods provided sufficient linearity and recovery with high precision. All methods are comparatively easy and inexpensive to conduct and allow the processing of many environmental samples because of fast, effective extractions. To our knowledge, this is the first target method set that allows for the analysis of multiple CUPs from different wild bee-related matrices in field-realistic small samples that can also be used for other flower-visiting insects. Our flower method (0.5 g) facilitates the study of CUPs in flowering plants using a small sample size that can be easily collected at a specific location within a limited amount of time. Although multiple pollen methods have already been established, we have developed a simple approach to analyse CUPs from the pollen of a single wild bee brood cell (0.1 g). Our unique method for extracting CUPs from individual wild bees (average dry weight female: 0.02 g, male: 0.01 g) provides a precise assessment of the exposure of wild bee individuals to CUP residues and allows to quantify the role of pesticide exposure in the observed decline in insects. Therefore, our methods enable straightforward and comprehensive analysis of the exposure of the CUP mixture to insect-relevant resources, as well as the insects themselves. The methods can be implemented in standardised monitoring and can be further developed to detect metabolites and other approved cups in future steps. The methods can be used together with the methods of soil and vegetation [28] to access CUP residues in environmental monitoring.

The application of our methods to environmental samples from an agricultural landscape revealed that flowers, pollen provisions, and new post-pupal bees contain CUP residues. The collected data provide more accurate insight into the realistic exposure of insects to CUP mixtures in agricultural areas. A meta-analysis showed that the additive effects of agrochemicals may underestimate the interactive effects of stressors on bee mortality [62]; thus, environmental risk assessment schemes fail to protect pollinators, although insects can be exposed to CUPs in various ways throughout their lives. The samples from the vegetable sites presented the most CUP residues. Therefore, insects living close to vegetable fields are potentially more exposed to CUP mixtures than to other crop types. Here, we show that insects are already exposed to CUPs before hatching and that adults start living in the agricultural landscape with a CUP burden that increases during their lifetime. Information on the presence of CUP residues in different exposure matrices for flower-visiting insects can be used to incorporate multiple pesticides into new environmental risk assessment or management approaches.

## Supplementary Information

Below is the link to the electronic supplementary material.

Supplementary file1 (XLSX 166 KB)

## Data Availability

Data is provided within the main text and the Electronic Supplementary Material (ESM).
